# Molecular Cloning and Characterization of the Calcineurin Subunit A from *Plutella xylostella*

**DOI:** 10.3390/ijms141020692

**Published:** 2013-10-15

**Authors:** Xi’en Chen, Yalin Zhang

**Affiliations:** Key Laboratory of Plant Protection Resources & Pest Management of Ministry of Education, Northwest A&F University, Yangling 712100, Shaanxi, China; E-Mail: chenpp2006@nwsuaf.edu.cn

**Keywords:** *Plutella xylostella*, calcineurin subunit A, catalytic domain, expression, phosphatase activity

## Abstract

Calcineurin (or PP2B) has been reported to be involved in an array of physiological process in insects, and the calcineurin subunit A (CNA) plays a central role in calcineurin activity. We cloned the *CNA* gene from *Plutella xylostella* (*Px*CNA). This gene contains an ORF of 1488 bp that encodes a 495 amino acid protein, showing 98%, and 80% identities to the CNA of *Bombyx mori*, and humans respectively. The full-length of *Px*CNA and its catalytic domain (CNA_1–341_, defined as *Px*CNα) were both expressed in *Escherichia coli*. Purified recombinant *Px*CNA displayed no phosphatase activity, whereas recombinant *Px*CNα showed high phosphatase activity with a *K*m of 4.6 mM and a *k*cat of 0.66 S^−1^ against pNPP. It could be activated at different degrees by Mn^2+^, Ni^2+^, Mg^2+^, and Ca^2+^. The optimum reaction pH was about 7.5 and the optimum reaction temperature was around 45 °C. An *in vitro* inhibition assay showed that okadaic acid (OA) and cantharidin (CTD) competitively inhibited recombinant *Px*CNα activity with the IC_50_ values of 8.95 μM and 77.64 μM, respectively. However, unlike previous reports, pyrethroid insecticides were unable to inhibit recombinant *Px*CNα, indicating that the *P. xylostella* calcineurin appears not to be sensitive to class II pyrethroid insecticides.

## 1. Introduction

Calcineurin, also known as protein phosphatase 2B or PP2B, is a Ca^2+^/CaM-dependent protein serine/threonine phosphatase (PSP) playing an essential role in numerous calcium-dependent intracellular biological processes, including neurodevelopment and memory, immune response, cardiac hypertrophy, signal transduction, and skeletal muscle development [1,2]. Calcineurin is composed of the catalytic subunit A (CNA) and the regulatory subunit B (CNB) [2]. CNA contains an *N*-terminal catalytic domain responsible for dephosphorylation of the substrates, and three non-catalytic domains [1]. The phosphatase domain of CNA is structurally similar to the catalytic subunit of other PSPs and shows the same pattern of metal ion coordination [3]. The three non-catalytic domains are identified as the CNB-binding domain (BBH), the CaM-binding domain (CBD) and the autoinhibitory domain (AI) from mapping of CNA using limited proteolysis and structural analysis [4]. Calcineurin is inactive alone and only gains full phosphatase activity upon association with Ca^2+^-calmodulin (Ca^2+^-CaM) [1].

To date, calcineurin has been suggested as playing a role in a diversity of physiological processes in insects. In *Drosophila*, calcineurin is most abundant in the early embryo indicating its involvement in embryonic development [5]. Two calcineurin heterosubunits isolated from the *Bombyx mori* pheromone gland are localized in the cytoplasm of pheromone-producing cells [6], and are found to participate in the signal transduction of pheromone biosynthesis activating neuropeptide (PBAN) [7]. It has been reported that calcineurin inhibits the process of synaptic vesicle endocytosis at nerve terminals in *Drosophila* larvae [8], is required for completion of meiosis [9] and flight muscle formation [10], and is even involved in sleep and memory regulation [11,12]. Calcineurin also mediates cAMP/PKA signaling pathways and activates V-ATPase in blowfly salivary glands [13]. However, few attempts have been made to characterize and uncover the functions of other insect calcineurins. In addition, no *in vitro* properties of any insect calcineurin have been reported.

In this study, we isolated the catalytic subunit of calcineurin from *Plutella xylostella*. Both the recombinant full-length and phosphatase domain of *P. xylostella* CNA (*Px*CNα) were expressed in *Escherichia coli* and purified to apparent homogeneity. Moreover, the *in vitro* properties of the recombinant protein were studied.

## 2. Results and Discussion

### 2.1. Cloning and Sequence Analysis of *Px*CNA

To date, *CNA* genes from several insect species have been identified [5,6]. Based on RT-PCR and RACE, we obtained the full-length of *Px*CNA, which contains a 1488 bp ORF that encodes a protein of 495 amino acids with the predicted molecular mass of 56,036 Da and theoretical isoelectric point of 6.04. The nucleotide sequence of *Px*CNA cDNA was deposited in the GenBank database under the accession number KF425265.

**Figure 1:**
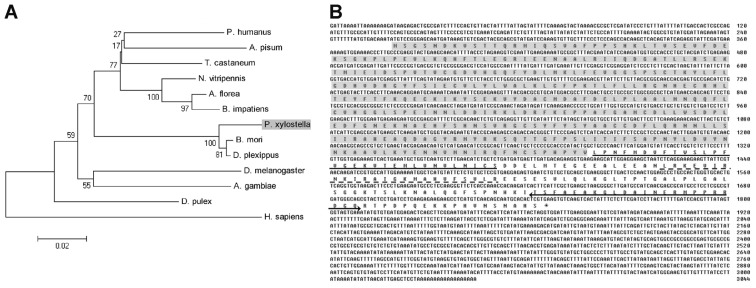
Sequence analysis of *Plutella xylostella* (*Px*CNA). (**A**) Phylogenetic analysis of *Px*CNA with other insect calcineurin subunit A (CNAs); (**B**) cDNA and deduced amino acid sequences of *Px*CNA. Phosphatase domain (gray shadow), CNB-binding domain (solid line), calmodulin-binding domain (broken line), and autoinhibitory domain (solid line with arrows) are indicated.

BLAST analysis of *Px*CNA deduced an amino acid sequence on NCBI that showed a strong similarity with CNAs from other insect species with the highest identity being 98% to the CNA of *B. mori*. It also shared an 80% identity to humans CNA. The phylogenetic tree showed CNA of *P. xylostella*, *B. mori*, and *D. plexippus* from Lepidoptera formed a small cluster (Figure 1A). Analysis of the amino acid sequence of *Px*CNA revealed that it contained a phosphatase domain at the *N*-terminus and its *C*-terminus consisted of three characteristic sequence motifs: a CNB-binding domain (BBH), a CaM-binding domain (CBD) and an autoinhibitory domain (AI) (Figure 1B).

### 2.2. Developmental Stage and Tissue-Specific Expression Patterns

**Figure 2:**
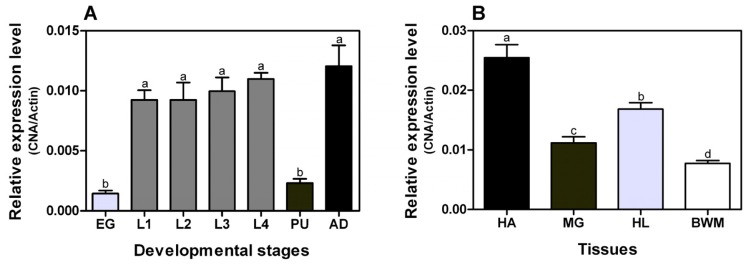
Expression analysis of *Px*CNA. (**A**) Relative expression levels in different developmental stages: eggs (EG); first-(L1), second-(L2); third-(L3); and fourth-instar (L4) larvae; pupae (PU) and adults (AD); (**B**) Relative expression levels in various tissues: head (HA); midgut (MG); haemolymph (HL) and body wall muscle (BWM). The actin gene (*Px*Actin) was used as the reference gene. Statistical differences were tested by ANOVA, and presented as a, b, c, and d.

Stage-specific expression patterns of the *P. xylostella CNA* gene were determined in eggs, four different larval instars (1st, 2nd, 3rd and 4th), pupae and adults by using Real-Time PCR. The *Px*CNA gene was expressed in all stages. Expression levels were relatively high in larval stages and adults, and lower in the eggs and pupae (Figure 2A). Expression levels of *Px*CNA in various tissues were also analyzed. As shown in Figure 2B, the *Px*CNA transcript had higher expression in head and was less abundant in body wall muscle. When *Drosophila* CNA was first isolated, it was undetectable at any stage of development by northern blot, suggesting it was expressed at very low levels [14]. In *B. mori*, the CNA transcript could be detected in various tissues with relative high levels in the head, anterior-middle silk gland and testis of fifth instar larvae, and relatively high levels in flight muscle, ovary, and the pheromone gland of newly eclosed adults [6]. So far, the mechanisms behind different levels of CNA expression in a variety of tissues and among different species are undefined.

### 2.3. Heterologous Expression and Purification of Recombinant Proteins

**Figure 3:**
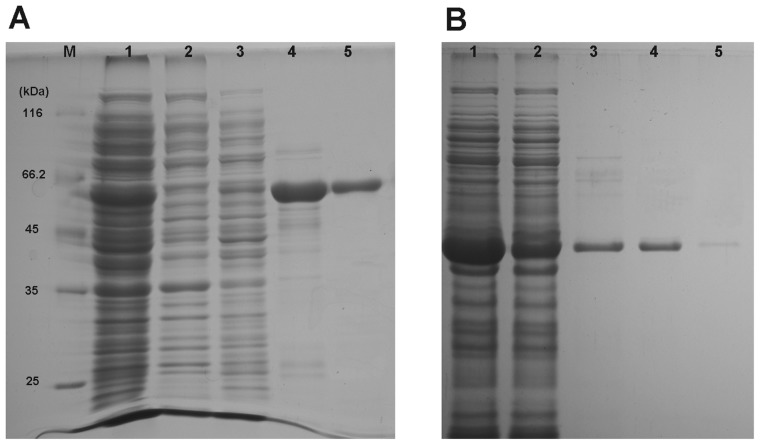
Purification of r*Px*CNA (**A**) and r*Px*CNα (**B**). M: Protein marker; 1, Soluble cell lysate; 2, Flow-through elution; 3, Wash-out elution; 4, 50 mM imidazole elution; 5, 150 mM imidazole elution.

To our knowledge, there is no report about the heterologous expression of insect CNA so far. We tried to express the *Px*CNA and *Px*CNα in *E. coli* expression system which has been used for producing recombinant mammalian CNAs successfully [15]. Recombinant *Px*CNA (r*Px*CNA) and recombinant *Px*CNα (r*Px*CNα) expressed in *E. coli* were detected as the apparent molecular weights of ~60 kDa and ~40 kDa on 12% SDA-PAGE (Figure 2), respectively, and this is close to the predicted protein sizes from the deduced amino acid sequences. Since the r*Px*CNA and r*Px*CNα proteins have been poly-histidine tagged, a Ni-NTA affinity column was used to purify target proteins in soluble form. The majority of r*Px*CNA was eluted by 150 mM imidazole (Figure 3A), while a high purity of r*Px*CNα was obtained in 50 mM imidazole (Figure 3B). After being dialyzed overnight at 4 °C, the protein concentrations were determined. Approximately 1.71 mg and 1.33 mg of highly purified r*Px*CNA and r*Px*CNα were achieved from a 500 mL cell culture, respectively.

### 2.4. Properties of r*Px*CNA and r*Px*CNα

**Figure 4:**
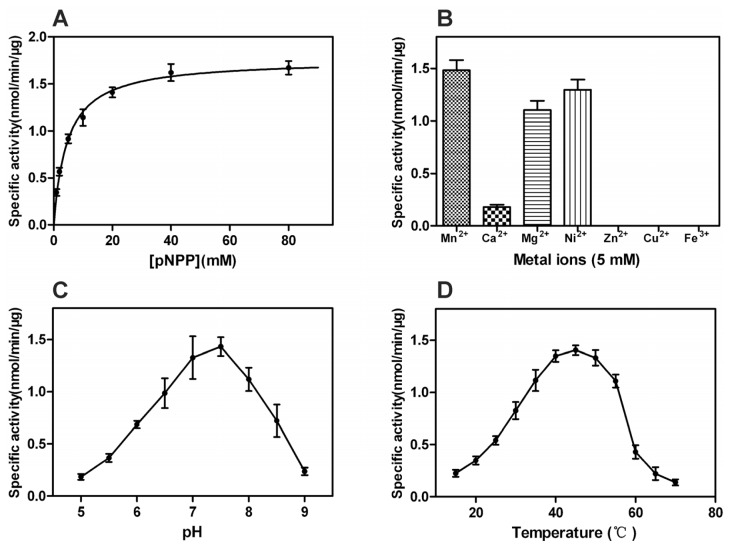
*In vitro* properties of r*Px*CNα. (**A**) Michaelis-Menten plot of r*Px*CNα toward pNPP; (**B**) Activation of r*Px*CNα phosphatase activity by different metal ions; (**C**) Optimum reaction pH curve; (**D**) Optimum reaction temperature curve. Each point is the mean ± S.D. of three independent experiments.

The phosphatase activities of r*Px*CNA and r*Px*CNα against pNPP were assayed. No phosphatase activity of r*Px*CNA was detected under our assay conditions, while r*Px*CNα displayed a high phosphatase activity. Enzyme-substrate reactions of Michaelis-Menten kinetics yielded a *Km* of 4.6 mM and a *Vmax* of 1.8 nmol/min/μg for r*Px*CNα using pNPP as substrate (Figure 4A). *kcat* and *kcat/Km* values were calculated as 0.66 S^−1^ and 143.48 M^−1^S^−1^, respectively. Because of the failure to acquire the phosphatase activity of r*Px*CNA, we did not determine its kinetics or other properties in this study. In most cases, the properties and function of calcineurin is directly determined by CNA, and the phosphatase domain plays a central role in CNA. CNA only exhibited full phosphatase activity binding with CNB and CaM in the presence of Ca^2+^ indicating the involvement of non-catalytic domains in the regulation of calcineurin [4]. Previous studies revealed that proteolytic cleavage of the auto-inhibitory and CaM-binding domains increased the native CNA phosphatase activity [4]. Increased phosphatase activity of CNA was also determined in the non-catalytic domain’s truncated recombinant CNA [15,16]. We found that, in the absence of CNB and CaM, r*Px*CNA had no phosphatase activity, whereas r*Px*CNα exhibited high phosphatase activity which is in agreement with previous reports.

The *in vitro* activation of native calcineurin, recombinant CNA and CNα dependent on the exogenous metal ions, such as Mn^2+^, Ni^2+^, Co^2+^, Mg^2+^, have been reported [17,18]. The phosphatase activity of r*Px*CNα was tested in the assaying buffer for different metal ions. Mn^2+^ was found to be the most effective activator of r*Px*CNα in our assay. Ni^2+^ and Mg^2+^ were 87.37% and 73.61%, respectively, as effective as Mn^2+^ in stimulation of r*Px*CNα. The activation of r*Px*CNα by Ca^2+^ was 12.22% as effective as by Mn^2+^. No activation by Zn^2+^, Cu^2+^ or Fe^3+^ was observed (Figure 4B). Consequently, the sequence of tested metal ions on r*Px*CNα activity stimulation was Mn^2+^ > Ni^2+^ > Mg^2+^ > Ca^2+^. Ca^2+^ was stated to be critical for the activity of calcineurin or CNA [1]; however, we found it has little effect on the activity of r*Px*CNα. Moreover, no activation effect was measured on recombinant rat CNα by Ca^2+^[17]. Our result with Zn^2+^, Cu^2+^, and Fe^3+^ exhibiting no activation on r*Px*CNα is consistent with a previous report [18], but the mechanism is still illusive. A previous study of direct metal ion binding demonstrated that Mn^2+^ and Ni^2+^ activated CNA activity mainly through binding at distinct metal ion binding sites on CNA [19]. Yet, these binding sites have not been identified. It was also implicated that metal ions activated CNA mainly via affecting the interaction between enzyme and substrate by having an effect upon the conformational changes from a native state to a hydrolysis state in an enzyme-ion-substrate complex [20]. More research is needed to explore this mechanism of exogenous metal ion activation of CNA.

The optimal reaction pH and temperature were estimated from a series of reactions in different pH assaying buffer and in pH 7.4 assaying buffer at different temperatures. The phosphatase activity of r*Px*CNα first increased and then decreased with ascending pH or temperature. The optimum reaction pH is about 7.5 and the optimum reaction temperature is around 45 °C (Figure 4C,D).

### 2.5. Effect of Pyrethroids and Phosphatase Inhibitors on r*Px*CNα

**Figure 5:**
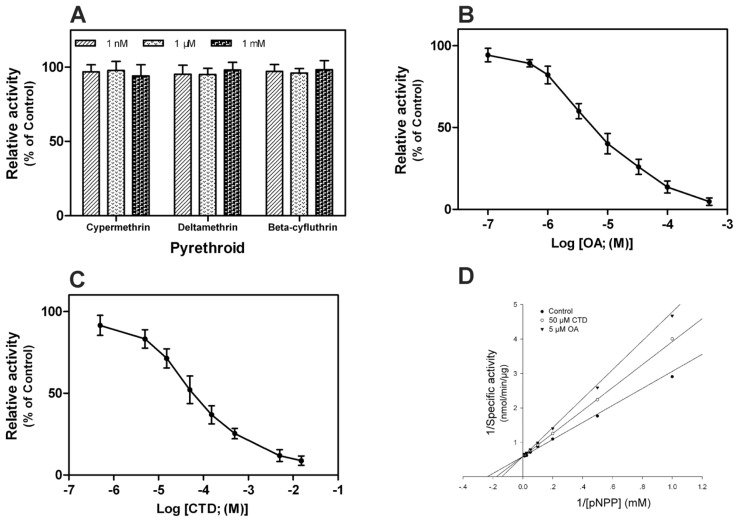
Inhibition assay of chemicals on r*Px*CNα. (**A**) Relative activity of r*Px*CNα in presence of 1 nM, 1 μM and 1 mM of pyrethroid insecticides; (**B**) Inhibition of r*Px*CNα by okadaic acid (OA); (**C**) Inhibition of r*Px*CNα by CTD; (**D**) Inhibition model of OA and cantharidin (CTD) on r*Px*CNα. Each point is the mean ± S.D. of three independent assays. The S.D. is omitted in panel **D**.

It was previously concluded that class II pyrethroid insecticides were potent inhibitors of bovine brain calcineurin with IC_50_ values of 10^−9^ to 10^−11^ M [21]. Three kinds of class II pyrethroid insecticides, cypermethrin, deltamethrin and beta-cyfluthrin, were investigated for their effect on r*Px*CNα. With insecticide concentrations of 2 nM, 2 μM and 2 mM, no inhibitory action was detected indicating these insecticides are not effective inhibitors of r*Px*CNA (Figure 5A). This discrepancy of our result with the previously published data might be explained as follows: (1) different sources for tested protein; (2) only the phosphatase domain of CNA was tested in this study; or (3) there were differences in experimental methodology. However, another independent inhibitory study showed that none of the class II pyrethroid insecticides were able to inhibit purified bovine or rat brain calcineurin. This supports the conclusion that they are not effective inhibitors of calcineurin [22]. Further investigation is needed to determine whether class II pyrethroid insecticides could inhibit insect calcineurin.

In our study, OA and CTD were found to be effective inhibitors of r*Px*CNα activity, with IC_50_ values of 8.95 μM and 77.64 μM, respectively (Figure 5B,C). An enzyme kinetics study in the presence of OA or CTD showed that both act on r*Px*CNα in a competitive inhibition mode with the *K*m decreasing and *Vmax* unchanged (Figure 4D). OA and CTD have been reported as potent inhibitors of PP1, PP2A, PP4 and PP5, but are relative weak inhibitors of calcineurin [23]. We found the IC_50_ values of these two chemicals on r*Px*CNα (OA, IC_50_ = 11.6 μM; CTD, IC_50_ = 58.7 μM) were higher than those on human calcineurin (OA, IC_50_ ~ 4 μM; CTD, IC_50_ > 10 μM) indicating that *P. xylostella* calcineurin may be less sensitive, in contrast to human calcineurin, to protein phosphatase inhibitors. However, what causes the affinity differences of OA or CTD toward human calcineurin and *P. xylostella* calcineurin needs to be investigated. Comparison of predicted three-dimensional structures of *P. xylostella* calcineurin obtained by homology modeling with resolved human calcineurin structure would be an alternative approach.

OA was found to bind to the hydrophobic groove on the surface of PP1 or PP2A occupying the active-site pocket [24,25], and CTD was reported binding to the active-site pocket of PP5 [26]. The results of enzymatic reactions with OA and CTD revealed that both competitively inhibit r*Px*CNα activity suggesting they bind the active site of r*Px*CNα. Thus, we propose that OA or CTD share the same binding modes in *P. xylostella* calcineurin with those in human protein phosphatases. Since specific inhibitors of enzymes have been proven to be a valuable tool to investigate the function of targeting proteins *in vivo* [27], we anticipate the use of other more-specific CNA inhibitors for studying the physiological roles of CNA in insects.

## 3. Experimental Section

### 3.1. Insects and Chemicals

Insecticide-susceptible strains of *P. xylostella* were reared at 25 ± 2 °C, 50% relative humidity and a photoperiod of 16L:8D, and fed on pakchoi cabbage.

OA and pNPP were purchased form Sigma Chemical Corporation (St. Louis, Missouri, USA). CTD was extracted from *Mylabris phalerata* (Chinese blister beetle) (purity >95%) and stored in our lab. Cypermethrin, deltamethrin and beta-cyfluthrin were purchased from Jingchun Co. Ltd., Shanghai, China. All other chemicals were of research grade or better and were obtained from commercial sources.

### 3.2. Cloning and Sequence Analysis of *Px*CNA

Total RNA was extracted from 4th-instar larvae using Trizol plus (TaKaRa, Dalian, China) according to manufacture’s instructions, and treated with DNase using a DNase I kit (Fermentas, Ontario, Canada). Then cDNA was synthesized using a First Strand cDNA Synthesis kit (Fermentas, Ontario, Canada). Degenerate primers were designed based on the CNA amino acid sequences from *Anopheles gambiae* (XP_321872), *Bombus impatiens* (XP_003492281), *B. mori* (NP_001037025), *D. melanogaster* (NP_523373), *Tribolium castaneum* (XP_968705) *Danaus plexippus* (EHJ68122), *Apis florae* (XP_003689692), *Nasonia vitripennis* (XP_001602102), *Pediculus humanus* (XP_002432635), *Acyrthosiphon pisum* (XP_001945831), and *Daphnia pulex* (EFX87567) by using two online tools, Blocks WWW Server (http://blocks.fhcrc.org/blocks/) and CODEHOP (http://bioinformatics.weizmann.ac.il/blocks/codehop.html). A pair of primers, a forward one: 5′-GCGACATCCACGGCCARTTYTAYGA-3′ and a reverse one: 5′-GGGGCAGGGCGCCNGTNGGNGT-3′, were used in PCR with first strand cDNA as the template under the following PCR conditions: 94 °C for 3 min, 35 cycles of 94 °C for 30 s, 60 °C for 30 s, 72 °C for 1 min, followed by final extension at 72 °C for 7 min. The purified fragment was inserted into pMD 19-T vector (TaKaRa, Dalian, China) and transformed into *E. coli* DH5α (TaKaRa, Dalian, China), then sequenced (AuGCT, Inc., Beijing, China).

Rapid amplification of cDNA ends (RACE) was conducted using the BD SMART^™^ RACE kit (Clontech, Palo Alto, CA, USA) following manufacturer’s instructions. 5′-RACE specific primer 5′-TCAGTCAGATGTCGGCACTCGTGG-3′ and 3′-RACE specific primer 5′-GCTGCAGCTG AAGGGCTTGACTCC-3′ were designed from the obtained *Px*CNA fragment. PCR conditions were as follows: 94 °C for 3 min, 35 cycles of 94 °C for 30 s, 60 °C for 30 s, 72 °C for 2 min, and final extension at 72 °C for 7 min. PCR products were sequenced as mentioned above.

The open reading frame (ORF) of *Px*CNA was predicted using ORF Finder (http://www.ncbi.nlm.nih.gov/gorf/gorf.html). Primers used to amplify the ORF were a sense primer 5′-CATATG*CACCACCACCACCACCAC*TCCGGGAGC AATGATAAAGTG-3′ with *Nde* I restriction site (underlined) and 6 histidine codons (italic), and an antisense primer 5′-AAGCTTTCACGAGTG TGCATTGCTGTTGAC-3′, containing the *Hind* III restriction site (underlined). PCR conditions were as follows: 94 °C for 3 min, 35 cycles of 94 °C for 30 s, 60 °C for 30 s, 72 °C for 1.5 min, followed by a final extension at 72 °C for 7 min. PCR products were sequenced as mentioned above.

The similarity analysis of deduced amino acid sequences was performed by using BLAST programs (http://blast.ncbi.nlm.nih.gov/Blast.cgi). The phylogenetic analyses were conducted by MEGA 5.0 (http://www.megasoftware.net/) by the maximum parsimony method using amino acid sequences of other insect CNAs and the human CNA (NP_001124163) as an outgroup. The ExPASy Compute pI/Mw tool (http://web.expasy.org/compute_pi/), was used to predict the molecular weight and isoelectric points of *Px*CNA.

### 3.3. Stage- and Tissue-Specific Expression of *Px*CNA

*P. xylostella* eggs, larvae of 1st instar to 4th instars, pupae and adults were collected. Various tissues, such as head, midgut, and body wall muscle, were dissected from the 4th instar larvae on ice. The haemolymph leaked out after puncturing the abdoments of the larvae, and was collected with a 20 μL pipette; then centrifuged at 12,000 × *g* for 30 min at 4 °C to remove haemocytes and cell debris, and the supernatant was stored at −80 °C until use. Total RNA isolation and cDNA synthesis were performed as described above. The forward primer, 5′-TCCAAGGGTTCTCACCAAAC-3′, and the reverse primer, 5′-GAGGATCAGGAGTACGCTGC-3′, were designed using Primer 3 (http://www.simgene.com/Primer3). The *P. xylostella* actin gene (accession: JN410820) was used as a reference for RT-PCR analysis, with the forward primer, 5′-GCGACTTGACCGACTACC-3′, and the reverse primer, 5′-GGAATGAGGGCTGGAACA-3′. Using 100-fold diluted cDNA as template, the Real-time quantitative PCR reactions were carried out in triplicate on a thermal cycler (iQ 5, Bio-Rad, Philadelphia, PA, USA) using the Ultra SYBR Mixture (CWBIO, Beijing China) according to the manufacture’s instructions. Thermal cycling conditions were 95 °C for 10 min, 40 cycles of 95 °C for 15 s, and 60 °C for 1 min, then followed by dissociation analysis to check the homogeneity of the PCR product. Relative transcript levels of *Px*CNA gene was determined by the 2^−Δ^*^Ct^* equation: 2^−[^*^Ct^*^tar −^*^Ct^*^ref]^ (*Ct*tar, *Ct* value of *Px*CNA gene; *Ct*ref, *Ct* value of *Px*Actin gene). The *Px*CNA data were expression as means ± stardard deviation (SD) and were analyzed by One-Way ANOVA.

### 3.4. *E. coli* Expression and Purification

Both the open reading frame (ORF) and phosphatase domain of *Px*CNA (*Px*CNα) with 6 histidine codons and *Nde* I site at *N*-terminal, and the *Hind III* site at the *C*-terminal, were cloned into *Nde* I and *Hind* III (TaKaRa, Dalian, China) double-digested pET43.1b expression vector (Novagen, Madison, WI, USA), and transformed into the *E. coli* strain BL21(DE3) (Novagen, Madison, WI, USA). Positive transformants were grown in LB medium at 37 °C until OD_600_ reached 0.6, then induced with 0.1 mM IPTG for 48 h at 18 °C.

Cells were collected by centrifugation at 7000 × *g* for 10 min at 4 °C. The pellet cells were re-suspended in Buffer A (20 mM Tris-HCl, pH = 8.0, 20 mM imidazole, 300 mM NaCl, 0.1% β-mercaptoethanol, 1.0 mg/mL iysozyme and 1 mM PMSF) and incubated for 30 min at room temperature followed by sonication for 5 min on ice; the debris was then pelleted by centrifugation (20,000 × *g* for 20 min at 4 °C). The soluble fraction was loaded onto a Buffer B (20 mM Tris-HCl, pH = 8.0, 20 mM imidazole, 300 mM NaCl) pre-equilibrated Ni-NTA affinity column (Transgen, Beijing, China) to bind the 6× His tagged protein; the non-specific proteins were washed out with Buffer B. The target protein was eluted with Buffer B containing serial concentrations of imidazole. Eluted solutions were subjected to 12% SDS-PAGE and stained with Coomassie brilliant blue. The target protein was dialyzed against Buffer C (Buffer B + 50% glycerol) overnight and stored at −20 °C. The protein concentration was assessed using the method of Bradford [28].

### 3.5. Phosphatase Activity Determination *In Vitro*

Phosphatase activity was determined using pNPP as the substrate as described by Wei and Li [29] with slight modification. The assay buffer contained 20 mM Tris, pH 7.4, 0.1% β-mercaptoethanol, 20 mM pNPP, 0.2 mg/mL BSA, and 0.5 mM MnCl_2_. Reactions were initiated after adding 1 μg of purified protein into the assaying buffer in a final volume of 200 μL and incubated at 35 °C for 20 min, then terminated with 1.8 mL 0.5 M Na_2_CO_3_, 20 mM EDTA. Control reactions were assayed without enzyme. The sample absorbance was measured at *A*_410_. Kinetic parameters of the enzyme were determined by using a Michaelis-Menten plot analysis of data obtained under the above assay conditions with pNPP concentrations of 1, 2, 5, 10, 20, 40, and 80 mM.

A metal ion activation assay was carried out in the presence of 0.5 mM of Mn^2+^, Ca^2+^, Mg^2+^, Cu^2+^, Ni^2+^, Zn^2+^ or Fe^3+^. The optimum reaction temperature was assayed at different temperatures and optimum reaction pH was determined in various pH substation solutions.

### 3.6. *In Vitro* Inhibition Assay

OA and CTD were dissolved in dimethyl sulphoxide (DMSO) to provide 10 mM and 100 mM stock solutions, respectively. Cypermethrin, deltamethrin and beta-cyfluthrin were dissolved in ethanol to give 50 mM stock solutions. All solutions were diluted to the desired concentrations with assay buffer before use. Before starting the reaction, the enzyme was incubated with the chemicals for 5 min at room temperature. A dose-response assay was used to determine the IC_50_. The non-enzyme reaction was taken as the background control, while the non-pyrethroid/inhibitor reaction was used as the full-activity control. An inhibition ratio was calculated as the percentage of *A*_410_ values of the inhibition assay reaction divided by the full-activity control, having subtracted the background control *A*_410_ value for both.

## 4. Conclusions

In summary, we isolated the CNA gene from *P. xylostella* and found that it was expressed in all developmental stages but the expression levels were relatively low. Both the full-length and phosphatase domain of *Px*CNA were expressed in *E. coli*. The recombinant *Px*CNA displayed no phosphatase activity; whereas the recombinant phosphatase domain (*Px*CNα) showed high phosphatase activity, which could be activated at different degrees by Mn^2+^, Ni^2+^, Mg^2+^, and Ca^2+^. Two protein phosphatase inhibitors, okadaic acid and cantharidin, strong inhibited recombinant *Px*CNα activity in a competitive mode. Three kinds of pyrethroid insecticides, which have been regarded as mammalian calaineurin/CNA inhibitors, exhibited no inhibitory effect on recombinant *Px*CNα, indicating the *P. xylostella* calcineurin/CNA appears insensitive to class II pyrethroid insecticides. This might be the first report of *in vitro* characterizations of heterologous expressed insect CNA.
